# Effect of polydopamine on bonding characteristics and color stability of blood contaminated Biodentine to composite

**DOI:** 10.3389/fdmed.2026.1945910

**Published:** 2026-09-11

**Authors:** Ihtisam Alam, Aravind R. Kudva, S. Vidhyadhara Shetty, Jayprakash Kukkila

**Affiliations:** 1 Department of Conservative Dentistry and Endodontics, Yenepoya Dental College,Yenepoya (Deemed to be University), Mangaluru, Karnataka, India; 2 Department of Dental Materials, Biomaterials and Research Center, Yenepoya (Deemed to be University) Dental College, Mangaluru, Karnataka, India

**Keywords:** biodentine, blood contamination, colour stability, polydopamine, resin composite, shear bond strength

## Abstract

**Introduction:**

Calcium silicate cements such as Biodentine are frequently placed in a blood-contaminated field during vital pulp therapy and must subsequently be bonded to an overlying resin composite. Polydopamine (PDA) improves the wettability and bond strength of mineral trioxide aggregate to resin composite, but its effect on blood-contaminated Biodentine is unknown.

**Aim:**

To determine the effect of PDA pretreatment on the shear bond strength (SBS), failure mode and colour change (*Δ*E) of blood-contaminated and uncontaminated Biodentine bonded to resin composite.

**Materials and methods:**

Seventy-six standardised Biodentine specimens (5 mm diameter × 3 mm) were prepared with blood contamination (present/absent) and PDA pretreatment (present/absent) as the two factors (*n* = 19 per cell). After adhesive application, bulk-fill composite build-up and 45 days of storage at 37 °C, SBS was measured, failure modes and *Δ*E was analysed.

**Results:**

Blood contamination significantly reduced SBS [F(1,72) = 18.86, *P* < 0.001, partial *η*^2^ = 0.21] and the contamination × PDA interaction was significant [F(1,72) = 7.39, *P* = 0.008, partial *η*^2^ = 0.09], whereas the main effect of PDA was not [F(1,72) = 2.07, *P* = 0.155]. Simple-effect analysis showed that PDA increased SBS in contaminated specimens (6.44 → 7.77 MPa; mean difference 1.33 MPa, 95% CI 0.43–2.23; *P* = 0.004) but not in uncontaminated specimens (8.70 → 8.29 MPa; *P* = 0.368). Without PDA, contamination lowered SBS by 2.26 MPa (*P* < 0.001), whereas after PDA pretreatment the difference between contaminated and uncontaminated specimens was no longer significant (*P* = 0.254). Contamination significantly increased *Δ*E (*P* < 0.001) whereas PDA did not (*P* = 0.102); all groups exceeded the clinical acceptability threshold. Failure-mode distribution differed significantly among groups (Fisher–Freeman–Halton exact *P* = 0.021; Cramér's V = 0.30), cohesive failure within Biodentine predominating in the contaminated groups.

**Conclusions:**

Within the limitations of this *in vitro* study, blood contamination occurring during setting reduced the shear bond strength of Biodentine to resin composite and increased its discolouration. PDA pretreatment significantly improved bond strength in contaminated specimens only, abolishing the statistically significant contamination penalty, and did so without aggravating colour change.

## 1 Introduction

Tricalcium silicate-based hydraulic cements have become indispensable in contemporary endodontics. Biodentine (Septodont, France) is widely used as a dentine substitute and bioactive repair material for direct and indirect pulp capping, pulpotomy, apexification, perforation repair and root-end filling, owing to its sealing ability, short setting time, favourable mechanical behaviour and capacity to induce hydroxyapatite formation and reparative dentinogenesis (1–4). In many of these clinical scenarios the freshly placed cement must subsequently be covered by a definitive resin composite restoration so that a single coronal seal protects the underlying pulp–dentine complex from bacterial microleakage.

The reliability of the Biodentine–composite interface therefore determines the long-term success of these restorations. Unfortunately, the interface is established under demanding intra-oral conditions. Haemostasis is frequently incomplete during pulp capping and pulpotomy, and the high-pH, hydrophilic, still-maturing surface of a calcium silicate cement is readily contaminated by blood. Blood components, principally fibrin and erythrocytes, are deposited onto and within the porous cement surface, physically obstructing micromechanical interlocking and chemically interfering with adhesive polymerisation. Several investigations have shown that blood contamination markedly reduces the bond strength of resin systems to dentine and to calcium silicate cements, and that it shifts the predominant failure pattern away from the substrate (5, 6).

Blood contamination of calcium silicate cements is also an aesthetic liability. Haemoglobin and its degradation products diffuse into the cement and undergo oxidation, producing the dark discolouration that has long been recognised with mineral trioxide aggregate (MTA) and, to a lesser degree, with Biodentine (7–9). Because Biodentine omits the bismuth oxide radiopacifier responsible for much of the discolouration of older MTA formulations, it is regarded as comparatively colour-stable; nevertheless, contact with blood can still drive its colour change (10, 11) beyond the clinically perceptible threshold (12, 13), an important consideration when the material is placed in the aesthetic zone or close to the cervical margin of an anterior tooth.

Strategies to rescue a compromised bonding substrate have therefore attracted considerable interest. Among emerging surface conditioners, polydopamine (PDA) is especially promising. Inspired by the 3,4-dihydroxy-L-phenylalanine (DOPA)-rich adhesive proteins that allow marine mussels to attach to wet surfaces, dopamine self-polymerises under mildly alkaline, aerobic conditions to deposit a thin, conformal, surface-adherent PDA film on virtually any substrate, including ceramics and hydraulic cements (14, 15). The catechol and amine functionalities of PDA establish covalent and non-covalent interactions with both the inorganic substrate and the resin adhesive, improving surface wettability and bridging an otherwise unfavourable interface. In dentistry, PDA-incorporated or PDA-pretreated systems have improved resin–dentine bond durability (15), and PDA pretreatment of MTA has been shown to increase its shear bond strength to resin composite while preserving surface integrity after etching (16).

Despite this progress, the behaviour of PDA on Biodentine — and, critically, on a Biodentine surface that has already been compromised by blood — has not been reported. If PDA could re-establish bonding to a blood-contaminated calcium silicate substrate without worsening discolouration, it would offer a simple, inexpensive chair-side intervention for a common clinical problem. The present *in vitro* study was designed to address this gap.

The aim of this study is to determine the effect of polydopamine pretreatment on the bonding characteristics (shear bond strength and failure mode) and the colour stability of blood-contaminated and uncontaminated Biodentine bonded to resin composite.

## 2 Materials and methods

### 2.1 Study design and reporting

This was a randomised *in vitro* experimental study with a balanced two-factor (2 × 2) fully crossed design. Factor 1 was blood contamination (present = Group I; absent = Group II) and Factor 2 was PDA pretreatment (present = subgroup A; absent = subgroup B), yielding four cells of equal size: IA (contaminated + PDA), IB (contaminated, no PDA), IIA (uncontaminated + PDA) and IIB (uncontaminated, no PDA). Specimens were allocated to the four cells by simple randomisation.

Ethical approval from the institutional review boards of Yenepoya Deemed to be University Dental College, Yenepoya Ethics Committee 2, YEC2/2026/081 was obtained on 07/05/2026, and the study was conducted in accordance with PRILE 2021 guidelines (17).

### 2.2 Sample size calculation

The sample size for the present study was calculated using G*Power software (version 3.1.9.7). The calculation is based on a power analysis for an ANOVA with four groups, assuming a large conventional effect size of 0.4, a 5% level of significance, and 80% power. The sample size is = 76 (19 in each group).

### 2.3 Synthesis of polydopamine

Polydopamine was prepared in the Department of Chemistry, National Institute of Technology Karnataka (NITK), Surathkal, India. Dopamine hydrochloride (20 mg) and Tris buffer powder (12.11 mg) were weighed on an electronic analytical balance and dispensed into a beaker containing 10 mL of distilled water. The mixture was stirred on a magnetic stirrer for 1 min to obtain a homogeneous solution and then left exposed to ambient air to permit oxidative self-polymerisation of dopamine (14); the progressive colour change of the solution to dark brown/black was taken as a visual indicator of PDA formation.

### 2.4 FTIR characterisation

**Figure 1:**
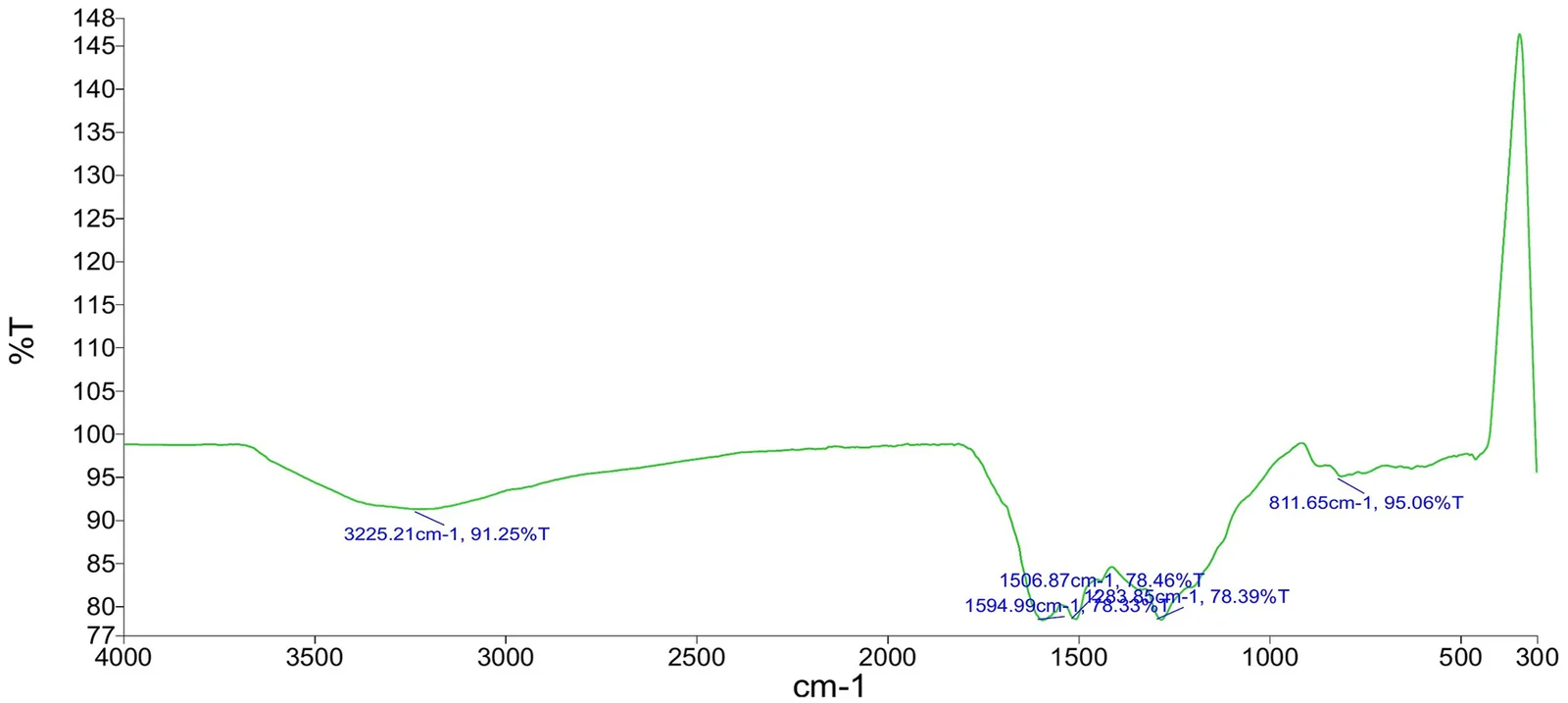
FTIR characterisation of polydopamine.

Polymerisation was confirmed by Fourier-transform infrared (FTIR) spectroscopy (PerkinElmer Spectrum IR). The spectrum was inspected for the characteristic absorption bands of PDA — broad O–H/N–H stretching, aromatic C = C and C = N stretching, and catechol C–O features — which, together with the visually observed colour transition, were used to verify adequate conversion of dopamine into PDA prior to its use as a surface conditioner. Interpretation was restricted to the 4,000–600 cm⁻^1^ region. Below approximately 600 cm⁻^1^ the trace lies at the low-wavenumber limit of the detector and returned an instrument quality-check warning, so this region was excluded from both the figure and the analysis. The spectrum was examined for the absorption bands characteristic of polydopamine, and assignments were made by reference to published polydopamine spectra (14, 15, 18). The spectrum is presented in Figure 1 and the resolved bands with their assignments in Table 1.

**Table 1:** ATR-FTIR absorption bands resolved for the synthesised polydopamine, with assignments.

Band (cm⁻^1^)	%T	Intensity	Assignment	Structural significance
3,225	91.25	Broad, medium	Overlapping O–H and N–H stretching	Catechol hydroxyl and indole/secondary amine N–H; the breadth of the envelope and its downward shift relative to crystalline dopamine hydrochloride indicate extensive hydrogen bonding within a polymeric network
1,595	78.33	Strong	Conjugated aromatic C = C ring stretching	Aromatic framework of the catechol and indole moieties retained after polymerisation
1,507	78.46	Strong	N–H scissoring of the nitrogen heterocycle, with aromatic ring contribution	Indole/indoline units generated by oxidative cyclisation of dopamine; resolution of the 1,595/1,507 cm⁻^1^ pair increases with degree of polymerisation
1,284	78.39	Strong	Phenolic (catechol) C–O stretching, with possible C–N contribution	Retention of the catechol functionality responsible for the substrate adhesion exploited in this study
812	95.06	Weak	Aromatic C–H out-of-plane bending	Substituted aromatic ring, consistent with indole-linked dihydroxy aromatic units

Band positions located by instrument peak picking; %T values are transmittance minima. Interpretation restricted to 4,000–600 cm⁻^1^ (Section 2.4). Assignments after references (14, 15, 18).

### 2.5 Specimen preparation and grouping

**Figure 2:**
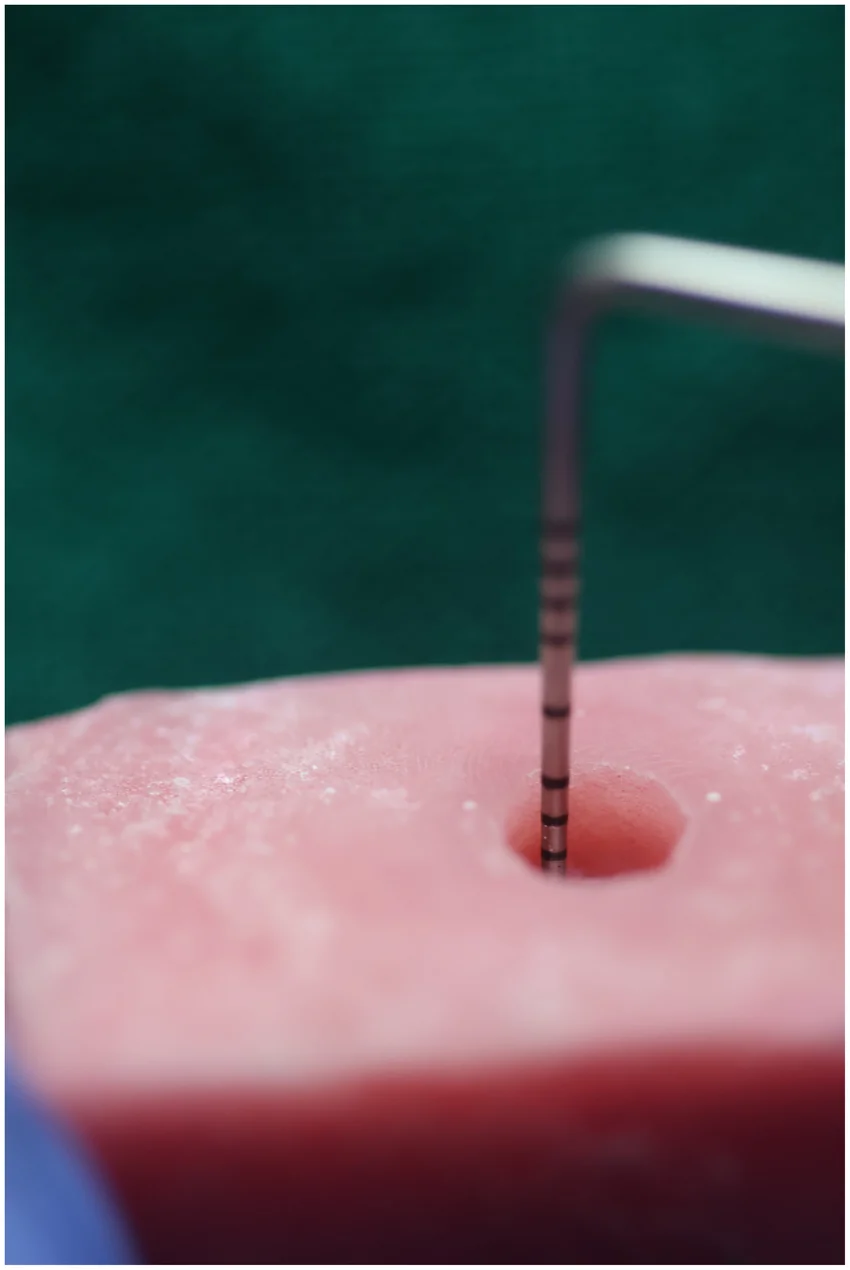
Depth of the cavity (3 mm).

A total of 76 acrylic molds were prepared with an inner diam­eter of 5 mm and height of 3 mm (Figure 2). They were randomly sorted into 2 groups; Group I: Blood contaminated and Group II: Uncontaminated Biodentine. Each group was further divided into 2 sub-groups depending on the surface pretreatment with of Biodentine with PDA. 5 mL blood was withdrawn from the investigator's median cubital vein using a 21G needle attached to a syringe by an experienced nurse. Then a drop of blood of volume approximztely 0.02 mL was placed in the base of the slots of each acrylic blocks belonging to the contaminated group just before cement placement. Biodentine was mixed according to the manufacturer's instructions and placed in the prepared acrylic slots with and without blood contamination. Then the samples were divided into two sub groups of 19 each, based on the surface pretreatment of Biodentine with PDA. Samples in groups 1a and IIa (PDA pretreatment), was coated with a layer of PDA prior to bonding with composite resin. 0.10 mL of PDA was dispensed on the surface of biodentine using a micro applicator tip for 15 s and left in place for 1 min. In groups Ib and IIb RC, it was directly bonded to the biodentine surface without an intermedi­ate layer of PDA.

### 2.6 Bonding and resin composite build up

**Figure 3:**
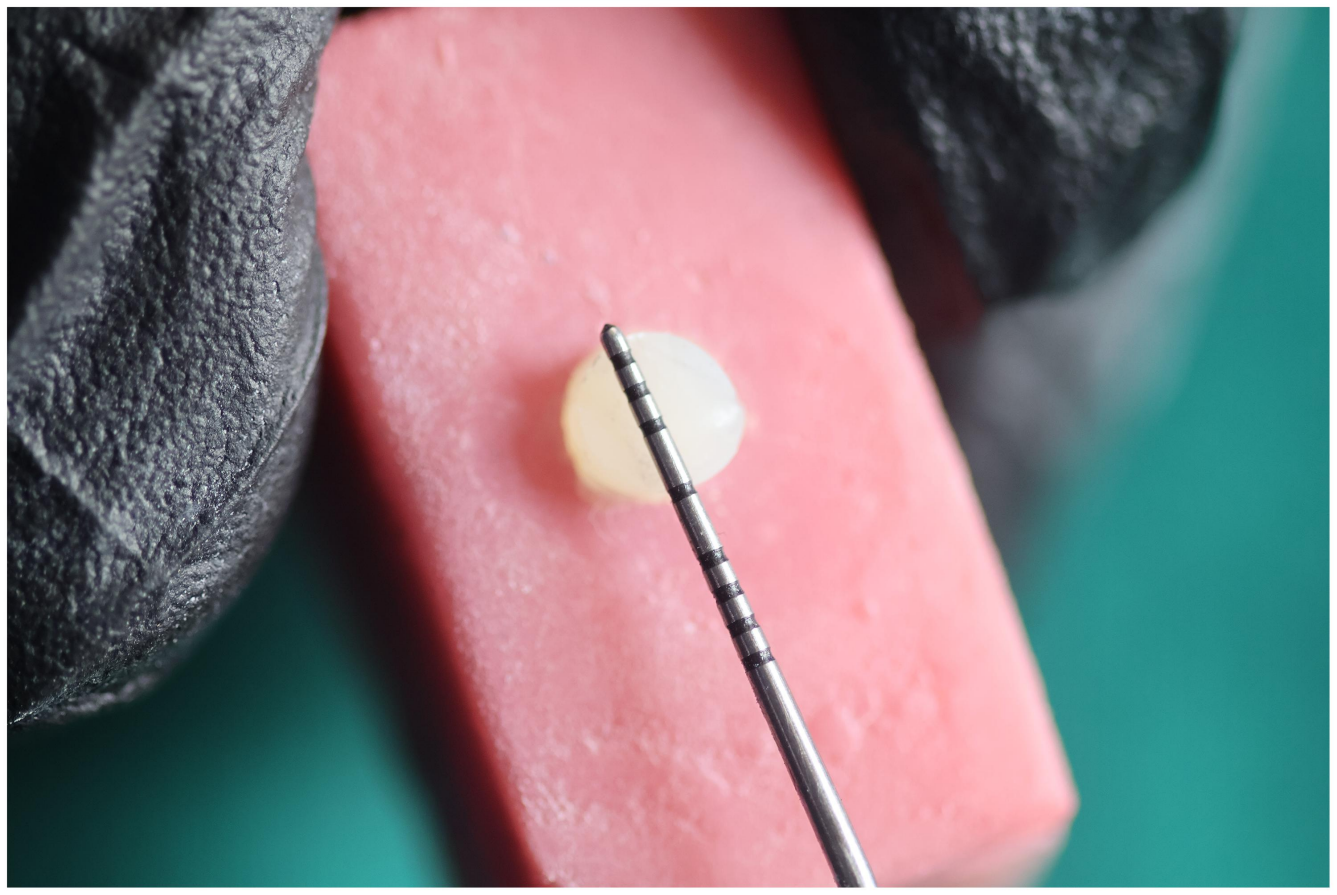
After placement of composite resin (Diameter-5 mm).

All the biodentine samples were bonded using a one-step self-etch (SE) adhesive system (Tetric N bond universal, Ivoclar). The adhesive was applied over the biodentine sur­face using an applicator tip. A cylindrical plastic transparent tube with an inner diameter of 5 mm and height of 2 mm was secured in place, prior to curing the adhesive with a light emitting diode (LED) light curing unit having an intensity of 1,000 mW/cm^2^ and the adhesive was cured for 10 s SDR plus was placed over the Biodentine surface, and cured for 20 s (Figure 3). The sam­ples were stored in 100% relative humidity at 37 °C for 45 days. This storage period is a deliberate, declared deviation from the 24 ± 2 h specified in ISO 29022:2013 (19).

### 2.7 Colour-change measurement

Colour was assessed with a clinical spectrophotometer (VITA Easyshade V). Baseline CIE L*a*b* values were recorded after setting, and final values after 45 days of incubation. The colour change (*Δ*E) was calculated as

*Δ*E = ([Lᵢ−L₀]^2^ + [aᵢ−a₀]^2^ + [bᵢ−b₀]^2^)^½^,

where *Δ*L represents brightness (black 0 to white 100), *Δ*a the red–green axis and *Δ*b the blue–yellow axis. Discolouration was regarded as clinically perceptible when *Δ*E ≥ 3.7 (12).

### 2.8 Shear bond strength testing

The SBS test was performed in a universal testing machine using a 50 kN load cell. A knife edge blade set at a crosshead speed of 0.5 mm/min was di­rected at the Biodentine-composite interface. SBS was calculated by dividing the peak load at failure with the sample surface area.

### 2.9 Analysis of failure mode

Failure modes of the four subgroups were analyzed by a single operator under the dental operating microscope and categorized as follows:

- Adhesive fracture: Fracture between restorative material and Biodentine.
- Cohesive fracture: Fracture within restorative material or Biodentine.
- Mixed fracture: Combination of cohesive and adhesive fracture

## 3 Statistical analysis

Data was coded in MS Excel and all statistical analysis were carried out using IBM SPSS 27 software. The summary of continuous variables were presented using mean, median, standard deviation, minimum, maximum, inter quartile (percentiles). Further, the continuous variables were assessed for Normality through the Shapiro–Wilk test. It is observed that 2 groups (IA,IB) do not follow Normality. Because the experiment was a balanced, fully crossed 2 × 2 factorial design, the primary analysis for SBS and for *Δ*E was a two-way analysis of variance (ANOVA) with blood contamination and PDA pretreatment as fixed factors, testing both main effects and their interaction. This model — rather than a one-way omnibus test across the four cells — is the analysis that corresponds to the stated study objectives, because only a factorial model can separate the effect of contamination, the effect of PDA and the dependence of one upon the other. Effect sizes are reported as partial *η*^2^ (0.01 small, 0.06 medium, 0.14 large).

Two-way ANOVA was retained as the primary model despite the departure from normality in two cells because the design is balanced with 19 specimens per cell, the ratio of the largest to the smallest cell variance was modest (1.41 for SBS and 1.67 for *Δ*E, both well below the conventional 3:1 heuristic), and the *F* test is robust to moderate non-normality under these conditions. As a sensitivity analysis, the factorial model was repeated after aligned rank transformation (ART), a non-parametric procedure that permits valid testing of main effects and interactions on ranked data (20), and the conclusions were unchanged; the results of the parametric model are therefore reported throughout.

Because a significant interaction was anticipated, four pre-planned simple-effect contrasts were specified for each outcome: the effect of PDA within each contamination stratum, and the effect of contamination within each pretreatment stratum. Contrasts were tested against the pooled error term of the factorial model (df = 72) and adjusted for multiplicity by the Holm procedure within each family of two contrasts. Mean differences are reported with 95% confidence intervals and Cohen's d.

Failure-mode distribution was cross-tabulated by group. Because four of the twelve expected cell counts were below five (minimum expected count 3.0), the asymptotic Pearson chi-square test was not appropriate and the Fisher–Freeman–Halton exact test was used as the primary test of association, with Cramér's V as the measure of effect size. The Pearson statistic is reported alongside for completeness only. Two-tailed *P* < 0.05 was considered statistically significant throughout.

## 4 Results

### 4.1 FTIR characterisation of polydopamine

The ATR-FTIR spectrum of the synthesised material is presented in Figure 1, and the resolved bands with their assignments are listed in Table 1. Five bands were identified within the interpretable region. A broad band of medium intensity was centred at 3,225 cm⁻^1^ (91.25%T), corresponding to overlapping O–H and N–H stretching. Three strong, closely spaced bands were resolved at 1,595 cm⁻^1^ (78.33%T), 1,507 cm⁻^1^ (78.46%T) and 1,284 cm⁻^1^ (78.39%T), attributable respectively to conjugated aromatic C = C ring stretching, to N–H scissoring of the nitrogen heterocycle of the indole/indoline unit, and to phenolic (catechol) C–O stretching. A weak band at 812 cm⁻^1^ (95.06%T) was consistent with aromatic C–H out-of-plane bending of a substituted ring. No discrete aliphatic C–H stretching band was resolved between 2,800 and 3,000 cm⁻^1^.

Three features of the spectrum support conversion of dopamine to polydopamine. First, the O–H/N–H envelope is broad and centred at 3,225 cm⁻^1^, appreciably below the sharper 3,320–3,350 cm⁻^1^ bands of crystalline dopamine hydrochloride; such a downward shift accompanied by broadening reflects extensive hydrogen bonding and the incorporation of N–H groups into a polymeric network. Second, the resolution of the paired bands at 1,595 and 1,507 cm⁻^1^ is the feature most consistently reported for the aromatic and heterocyclic ring system of polydopamine, and these two bands become better resolved as the degree of polymerisation increases. Third, the persistence of the catechol C–O band at 1,284 cm⁻^1^ confirms that the catechol functionality responsible for surface adhesion — the property on which this study depends — was retained. Together with the visually observed transition of the solution to dark brown/black, these findings are consistent with oxidative self-polymerisation of dopamine to polydopamine (14, 15, 18).

### 4.2 Shear bond strength

Descriptive statistics are given in Table 2. Mean SBS increased in the order IB (blood-contaminated, no PDA) < IA (blood-contaminated + PDA) < IIA (uncontaminated + PDA) < IIB (uncontaminated, no PDA), with means of 6.44, 7.77, 8.29 and 8.70 MPa respectively.

**Table 2:** Descriptive statistics for shear bond strength (MPa) by group (*n* = 19 per group).

Group	Mean	SD	Median	Min	Max	25th	75th
IA (contaminated + PDA)	7.77	1.53	8.00	4.34	11.44	6.79	8.41
IB (contaminated, no PDA)	6.44	1.36	6.59	3.83	9.67	6.11	7.07
IIA (uncontaminated + PDA)	8.29	1.29	8.21	6.25	10.20	7.28	9.23
IIB (uncontaminated, no PDA)	8.70	1.39	8.78	5.92	11.03	8.06	9.55

The two-way ANOVA (Table 3) showed a large, statistically significant main effect of blood contamination [F(1,72) = 18.86, *P* < 0.001, partial *η*^2^ = 0.208]: averaged across pretreatment, contaminated specimens bonded 1.39 MPa more weakly than uncontaminated specimens (7.11 versus 8.50 MPa). The main effect of PDA pretreatment was not significant [F(1,72) = 2.07, *P* = 0.155, partial *η*^2^ = 0.028], reflecting the fact that PDA acted in opposite directions in the two substrates and so had little average effect. Critically, the contamination × PDA interaction was significant [F(1,72) = 7.39, *P* = 0.008, partial *η*^2^ = 0.093], indicating that the effect of PDA depended on whether the substrate had been contaminated. The second and third null hypotheses were therefore rejected in respect of the interaction, and the first in respect of contamination.

**Table 3:** Two-way (2 × 2) analysis of variance for shear bond strength.

Source	Sum of squares	df	Mean square	F	P	Partial *η*^2^
Blood contamination	36.71	1	36.71	18.86	<0.001	0.208
PDA pretreatment	4.02	1	4.02	2.07	0.155	0.028
Contamination × PDA	14.38	1	14.38	7.39	0.008	0.093
Error	140.16	72	1.95	—	—	—
Corrected total	195.27	75	—	—	—	—

The pre-planned simple-effect contrasts (Table 4) localise the interaction. Within the contaminated substrate, PDA pretreatment raised SBS by 1.33 MPa (95% CI 0.43 to 2.23), a relative increase of 20.7% that was statistically significant [t(72) = 2.94, *P* = 0.004; Holm-adjusted *P* = 0.009] and corresponded to a large effect (d = 0.95). Within the uncontaminated substrate, PDA produced a small, non-significant reduction of 0.41 MPa (95% CI −1.31 to 0.49; *P* = 0.368). Viewed from the other direction, blood contamination reduced SBS by 2.26 MPa in the absence of PDA (95% CI −3.16 to −1.36; *P* < 0.001; d = −1.62), whereas after PDA pretreatment the contaminated and uncontaminated groups no longer differed significantly (mean difference −0.52 MPa, 95% CI −1.42 to 0.38; *P* = 0.254). PDA pretreatment therefore abolished the statistically detectable penalty imposed by blood contamination, although the point estimate remained below the uncontaminated control.

**Table 4:** Pre-planned simple-effect contrasts for shear bond strength (pooled error term, df = 72).

Contrast	Mean diff. (MPa)	95% CI	t	P	Holm-adj. P	Cohen's d
Effect of PDA, contaminated (IA−IB)	+1.33	0.43 to 2.23	2.94	0.004	0.009	0.95
Effect of PDA, uncontaminated (IIA−IIB)	−0.41	−1.31 to 0.49	−0.91	0.368	0.368	−0.29
Effect of contamination, no PDA (IB−IIB)	−2.26	−3.16 to −1.36	−4.99	<0.001	<0.001	−1.62
Effect of contamination, with PDA (IA−IIA)	−0.52	−1.42 to 0.38	−1.15	0.254	0.254	−0.37

### 4.3 Colour change (*Δ*e)

Descriptive statistics for *Δ*Eab are given in Table 5. All four groups exhibited a mean *Δ*Eab above the clinical acceptability threshold of 3.7 and, a fortiori, above the more conservative 50:50% acceptability threshold of 2.7. The greatest discolouration occurred in the contaminated group without PDA (IB, 5.74), followed by the contaminated group with PDA (IA, 5.30); the uncontaminated groups discoloured less (IIA 4.88; IIB 4.91).

**Table 5:** Colour change (*Δ*Eab) after 45 days by group (*n* = 19 per group).

Group	Mean *Δ*Eab	SD	Exceeds clinical acceptability threshold (*Δ*Eab = 3.7)
IA (contaminated + PDA)	5.30	0.62	Yes
IB (contaminated, no PDA)	5.74	0.71	Yes
IIA (uncontaminated + PDA)	4.88	0.55	Yes
IIB (uncontaminated, no PDA)	4.91	0.58	Yes

The two-way ANOVA (Table 6) showed a large, significant main effect of blood contamination [F(1,72) = 19.44, *P* < 0.001, partial *η*^2^ = 0.213], with contaminated specimens discolouring by 0.63 *Δ*E units more than uncontaminated specimens on average. Neither the main effect of PDA [F(1,72) = 2.75, *P* = 0.102, partial *η*^2^ = 0.037] nor the contamination × PDA interaction [F(1,72) = 2.09, *P* = 0.153, partial *η*^2^ = 0.028] reached significance. Blood contamination was therefore the dominant determinant of colour change, and PDA pretreatment did not aggravate discolouration.

**Table 6:** Two-way (2 × 2) analysis of variance for colour change (*Δ*Eab), with pre-planned simple-effect contrasts.

Source	Sum of squares	df	Mean square	F	P	Partial η^2^
Blood contamination	7.42	1	7.42	19.44	< 0.001	0.213
PDA pretreatment	1.05	1	1.05	2.75	0.102	0.037
Contamination × PDA	0.80	1	0.80	2.09	0.153	0.028
Error	27.49	72	0.38	—	—	—
Corrected total	36.76	75	—	—	—	—

Simple-effect contrasts (pooled error, df = 72; Holm-adjusted within each family of two): effect of PDA in contaminated specimens −0.44 *Δ*E units (95% CI −0.84 to −0.04; *P* = 0.031; Holm-adjusted *P* = 0.063); effect of PDA in uncontaminated specimens −0.03 (95% CI −0.43 to 0.37; *P* = 0.881). Effect of contamination without PDA +0.83 (95% CI 0.43 to 1.23; *P* < 0.001); effect of contamination with PDA +0.42 (95% CI 0.02 to 0.82; *P* = 0.040).

Within the contaminated substrate, specimens pretreated with PDA discoloured marginally less than untreated specimens (−0.44 *Δ*E units); this contrast was nominally significant but did not survive adjustment for multiplicity (Holm-adjusted *P* = 0.063) and, at less than half of a perceptibility threshold unit, is of no clinical consequence. It is reported as an exploratory observation only.

### 4.4 Failure-mode analysis

The distribution of failure modes is given in Table 7. Cohesive failure within the Biodentine was the predominant mode in the blood-contaminated groups (IA 58%; IB 63%), whereas adhesive failure became progressively more common as substrate quality improved, predominating in the uncontaminated group without PDA (IIB 63%). No mixed failures were recorded in group IIB.

**Table 7:** Distribution of failure modes by group, *n* (% within group).

Failure mode	IA	IB	IIA	IIB	Total
Adhesive	4 (21%)	3 (16%)	8 (42%)	12 (63%)	27 (36%)
Cohesive (within Biodentine)	11 (58%)	12 (63%)	7 (37%)	7 (37%)	37 (49%)
Mixed	4 (21%)	4 (21%)	4 (21%)	0 (0%)	12 (16%)
*Total*	19	19	19	19	76

Fisher–Freeman–Halton exact test: *P* = 0.021; Cramér's V = 0.301. Pearson *χ*^2^ = 13.76, df = 6, *P* = 0.032 (reported for completeness; four expected counts < 5).

Four of the twelve expected cell counts fell below five (minimum expected count 3.0), so the Fisher–Freeman–Halton exact test was used as the primary analysis. The association between group and failure mode was statistically significant (exact two-tailed *P* = 0.021), with a moderate effect size (Cramér's V = 0.301). The asymptotic Pearson statistic is reported for completeness (*χ*^2^ = 13.76, df = 6, *P* = 0.032) but was not relied upon.

## 5 Discussion

The present study evaluated, for the first time, the combined effect of blood contamination and polydopamine pretreatment on the bonding and colour behaviour of Biodentine bonded to resin composite. The null hypothesis was rejected: both blood contamination and PDA pretreatment influenced the measured outcomes. The principal findings were that blood contamination depressed shear bond strength and shifted failure toward cohesive fracture within the cement, that PDA pretreatment partially recovered the bond strength of contaminated Biodentine, and that PDA did not aggravate — and may have slightly mitigated — the discolouration caused by blood.

The reduction in bond strength of the contaminated, untreated group (IB) is consistent with the established literature. Dülger and colleagues reported that blood contamination significantly lowered the SBS of Biodentine to resin composite and shifted failure toward the adhesive interface (5), and Shalabi and co-workers demonstrated that blood contamination during setting impaired both the bond strength and the apatite-forming bioactivity of Biodentine (6). Blood proteins, particularly fibrin, are thought to occlude the surface porosity of the cement and to form a weak, contaminated boundary layer that interferes with adhesive infiltration and polymerisation, reducing both micromechanical interlocking and chemical interaction.

The partial recovery of bond strength in the contaminated PDA group (IA) is the most clinically relevant result. It mirrors the improvement that Keerthivasan and colleagues observed when PDA pretreatment increased the SBS of MTA to resin composite (16, 21). The catechol and amine groups of PDA are able to displace or integrate with the contaminated surface layer, re-establish surface wettability, and present reactive functional groups that bridge the inorganic cement and the resin adhesive. In the present study this bridging effect appears to have been most valuable precisely where the substrate was compromised: PDA raised the contaminated SBS toward the level of the uncontaminated groups, while adding little to the already favourable uncontaminated surface (IIA versus IIB). This substrate-dependent benefit is itself an important observation, because it suggests that PDA functions as a rescue conditioner rather than as a universal bond enhancer.

The failure-mode data support this interpretation. The contaminated groups failed predominantly by cohesive fracture within the Biodentine, indicating that the adhesive interface was, paradoxically, no longer the weakest link once the bulk cement had been weakened by blood incorporation; the uncontaminated, untreated group failed predominantly at the adhesive interface, the pattern expected when the cement itself is sound. The statistically significant association between group and failure mode reinforces the conclusion that contamination and surface conditioning alter not only the magnitude of the bond but also its mechanism of failure.

With respect to colour, all groups exceeded the perceptibility threshold of *Δ*E ≥ 3.7, with the contaminated groups discolouring most. This agrees with spectrophotometric studies showing that blood drives the colour change of calcium silicate cements above clinically perceptible levels, and that Biodentine, while more colour-stable than bismuth-containing MTA, is not immune to blood-induced discolouration (7, 8, 10, 11). Importantly, PDA pretreatment did not worsen discolouration despite being a melanin-like pigment, most likely because the deposited film is extremely thin and is itself overlaid by the adhesive and composite. From a clinical standpoint this is reassuring: the bonding benefit of PDA does not appear to carry an aesthetic penalty under the conditions tested.

### 5.4 Clinical implications

If confirmed, the practical implication is narrow but useful. When Biodentine has been placed into a field in which haemostasis was incomplete, a brief chair-side application of a polydopamine solution before adhesive application may recover a clinically meaningful proportion of the bond strength lost to contamination, at negligible cost and without an aesthetic penalty. Conversely, there is no evidence from these data to support routine PDA pretreatment of an uncontaminated Biodentine surface, where it conferred no benefit. The absolute SBS values in all groups (6.4–8.7 MPa) remain within the range commonly reported for calcium silicate cement–composite interfaces and below that of resin–dentine bonds, so the clinical priority remains adequate haemostasis rather than salvage.

### 5.5 Limitations

The following limitations should be borne in mind when interpreting these findings.

● This was an ***in vitro*** study. Specimens were prepared in acrylic moulds rather than in prepared cavities in human teeth, so the geometry, C-factor, substrate compliance, dentinal fluid pressure and pulpal environment of the clinical situation were not reproduced.
● The **contamination model** simulated blood present at the moment of cement placement, with consequent incorporation of blood into the bulk of the setting cement. Surface contamination of already-set Biodentine — the other clinically relevant scenario — was not modelled, and the two are not interchangeable.
● Only **one adhesive system** (a one-step self-etch universal adhesive) and **one composite** (a bulk-fill flowable) were evaluated. Results may differ with etch-and-rinse or selective-etch protocols, with other universal adhesives, or with conventional sculptable composites.

## 6 Conclusions

Within the limitations of this *in vitro* study, blood contamination occurring during the setting of Biodentine significantly reduced its shear bond strength to resin composite, shifted failure toward cohesive fracture within the cement, and increased discolouration beyond the clinical acceptability threshold.

The effect of polydopamine pretreatment on bond strength was significantly dependent on substrate condition. In blood-contaminated specimens, PDA pretreatment significantly increased shear bond strength and abolished the statistically detectable penalty imposed by contamination; in uncontaminated specimens it conferred no benefit. PDA did not aggravate colour change.

Polydopamine therefore shows promise as a simple, inexpensive chair-side rescue conditioner for calcium silicate substrates compromised by blood, but not as a routine surface treatment for sound substrates. Confirmation in aged specimens, on set-then-contaminated surfaces, and with direct characterisation of the polydopamine layer is required before clinical recommendations can be made.

## Data Availability

The original contributions presented in the study are included in the article/Supplementary Material, further inquiries can be directed to the corresponding authors.
